# Bayesian approach for sample size determination, illustrated with Soil Health Card data of Andhra Pradesh (India)

**DOI:** 10.1016/j.geoderma.2021.115396

**Published:** 2022-01-01

**Authors:** D.J. Brus, B. Kempen, D. Rossiter, Balwinder-Singh, A.J. McDonald

**Affiliations:** a Biometris, Wageningen University and Research, PO Box 16, Wageningen 6700 AA, Netherlands; b ISRIC – World Soil Information, PO Box 353, Wageningen 6700 AJ, The Netherlands; c Section of Soil & Crop Sciences, New York State College of Agriculture & Life Sciences, Cornell University, Ithaca, NY 14853, USA; d International Maize and Wheat Improvement Centre (CIMMYT), National Agricultural Science Centre Complex (NASC) Dev Prakash Shastri Marg New Delhi, G2, B Block, Delhi 110012, India

**Keywords:** Pedometrics, Soil fertility, Mixed Bayesian-likelihood approach, Frequentist approach, Design parameters, Credible interval

## Abstract

A crucial decision in designing a spatial sample for soil survey is the number of sampling locations required to answer, with sufficient accuracy and precision, the questions posed by decision makers at different levels of geographic aggregation. In the Indian Soil Health Card (SHC) scheme, many thousands of locations are sampled per district. In this paper the SHC data are used to estimate the mean of a soil property within a defined study area, e.g., a district, or the areal fraction of the study area where some condition is satisfied, e.g., exceedence of a critical level. The central question is whether this large sample size is needed for this aim. The sample size required for a given maximum length of a confidence interval can be computed with formulas from classical sampling theory, using a prior estimate of the variance of the property of interest within the study area. Similarly, for the areal fraction a prior estimate of this fraction is required. In practice we are uncertain about these prior estimates, and our uncertainty is not accounted for in classical sample size determination (SSD). This deficiency can be overcome with a Bayesian approach, in which the prior estimate of the variance or areal fraction is replaced by a prior distribution. Once new data from the sample are available, this prior distribution is updated to a posterior distribution using Bayes’ rule. The apparent problem with a Bayesian approach prior to a sampling campaign is that the data are not yet available. This dilemma can be solved by computing, for a given sample size, the predictive distribution of the data, given a prior distribution on the population and design parameter. Thus we do not have a single vector with data values, but a finite or infinite set of possible data vectors. As a consequence, we have as many posterior distribution functions as we have data vectors. This leads to a probability distribution of lengths or coverages of Bayesian credible intervals, from which various criteria for SSD can be derived. Besides the fully Bayesian approach, a mixed Bayesian-likelihood approach for SSD is available. This is of interest when, after the data have been collected, we prefer to estimate the mean from these data only, using the frequentist approach, ignoring the prior distribution. The fully Bayesian and mixed Bayesian-likelihood approach are illustrated for estimating the mean of log-transformed Zn and the areal fraction with Zn-deficiency, defined as Zn concentration <0.9 mg kg ^−1^, in the thirteen districts of Andhra Pradesh state. The SHC data from 2015–2017 are used to derive prior distributions. For all districts the Bayesian and mixed Bayesian-likelihood sample sizes are much smaller than the current sample sizes. The hyperparameters of the prior distributions have a strong effect on the sample sizes. We discuss methods to deal with this. Even at the mandal (sub-district) level the sample size can almost always be reduced substantially. Clearly SHC over-sampled, and here we show how to reduce the effort while still providing information required for decision-making. R scripts for SSD are provided as supplementary material.

## Highlights

• Sample Size Determination (SSD) is a crucial step in sampling design.
• Bayesian, mixed Bayesian-likelihood (MBL) and frequentist SSD approaches compared.
• Bayesian and MBL SSD approach account for uncertainty about design parameters.
• Various SSD criteria derived from probability distribution of credible intervals .
• Legacy data on Zn concentration in soil used for postulating prior distributions.

## 1 Introduction

This research was motivated by the desire to evaluate the sampling efficiency of the nationally-mandated Soil Health Card (SHC) Scheme in India. This scheme specifies soil sampling at a very high density every two years. For example, Andhra Pradesh (AP) state (162,975 ${\mathrm{km}}^{2})$ in Cycle 2 (2017/18–2018/19) recorded 2,393,8875 observations, a density of 14.7 ${\mathrm{km}}^{-1}$, or one per 6.8 ha. This is consistent with the SHC policy of one soil sample per 10 ha in rainfed and one per 2.5 ha in irrigated areas. Due to the large scope of the programme and the logistic challenges, not all observations are valid: duplicated records of the same observation (e.g. the 2.4 M SHC records generated in Cycle 2 in AP were generated from samples taken at 1.6 M unique geographic locations), coordinates of sampling locations are not agreeing with recorded administrative unit (district/mandal), or data values outside permissible ranges. Sampling locations are not necessarily revisited in subsequent sampling rounds.

The SHC data are used for soil fertilization recommendations at the field level. The very high sampling density, however, results in operational barriers. Staff time to collect mandated soil samples is often insufficient and laboratory throughput capacity insufficient for the task of ensuring timely high-quality analysis. Costs are high: an estimated USD 85 M. The question is whether this high investment in soil survey is cost-efficient. Would a reduction of the number of sampling locations also suffice, and perhaps increase the quality and consistency of the sampling effort?

To adapt an existing sampling design, the aim or aims of the survey must be made explicit, as well as the constraints in terms of the budget or the required quality of the survey results (de Gruijter et al., 2006). For instance, when the data are used for estimating a population mean, e.g. the mean of the residual nitrate concentration in the topsoil of a selected district in Andhra Pradesh, how precise should this estimated mean be? Or, if the data are used for mapping the Zn concentration in the topsoil of Andhra Pradesh, how precise (i.e., at each mapped location how close to the true value) should this map be? What precision level is needed for decision making?

The Soil Health Card scheme aims at answering several research questions. The SHC scheme was originally designed to address agricultural production challenges at field-scale but the data are also used to develop soil information at the level of an administrative unit^1^ within a state (e.g., districts or mandals) for policy support.

In this paper we focus on estimating the current status of soil fertility parameters at the level of districts and mandals. So the main question is how many sampling locations should be sampled in order to estimate the spatial means of soil fertility parameters of the various districts, given a precision requirement on these estimated means, for instance in terms of the standard error or the length of a 95% confidence interval. This type of information is particularly relevant for soil fertilization programmes at the level of the district. Think, for instance, of whether or not a fertilizer blend with Zn should be applied in a district.

Two fundamentally different approaches can be followed to decide on the sample size: the frequentist approach and the Bayesian approach (Adcock, 1997, Lindley, 1997). In the frequentist approach the sample size required for a given quality constraint is computed from a prior estimate of the population variance or, in case of estimating an areal fraction, a prior estimate of this areal fraction. For instance, given a chosen maximum length of a (1-α)-confidence interval of the population mean or the areal fraction, the smallest sample size is determined that results in an interval length that does not exceed the chosen length. In practice we are always uncertain to some extent about the design parameter (population variance, areal fraction). In case of an areal fraction this is even evident, otherwise no additional sampling would be needed to estimate this parameter. An important drawback of the frequentist approach for sample size determination (SSD) is that our uncertainty about the design parameter is not accounted for.

In the Bayesian approach for SSD the uncertainty about the design parameter is explicitly accounted for, by postulating a probability distribution which reflects our belief about what value the design parameter could be. There is extensive literature on Bayesian SSD in the statistical literature, see for instance Adcock, 1988, Joseph et al., 1995, Joseph and Bélisle, 1997, Pham-Gia, 1997, Wang and Gelfand, 2002, Pezeshk, 2003, M’Lan et al., 2008, Cao et al., 2009, Pezeshk et al., 2009, Brutti et al., 2014.

Bayesian SSD is commonly applied in clinical trials, see for instance Stallard, 1998, O’Hagan and Stevens, 2001, Gajewski and Mayo, 2006 to mention a few. To the best of our knowledge a Bayesian approach to determine a sample size is not applied yet in soil science. The aim of this paper is to explain in detail Bayesian SSD, and to illustrate this with the Soil Health Card survey in Andhra Pradesh, India.

## 2 Theory

### 2.1 Frequentist versus Bayesian approach

Two major schools of statistical thinking are termed frequentist and Bayesian. In the first approach, probability distributions are defined as frequency distributions in the long run. For instance, the probability distribution of the estimated population mean for a given random sampling design is equal to the frequency distribution of the estimated population mean if we repeat the selection of samples with this design an infinite number of times. The population parameters are considered fixed but unknown; we sample to estimate these.

In the Bayesian approach, probability has a fundamentally different meaning. A probability distribution of a population mean, for instance, expresses what we believe the population can be. In the Bayesian approach a probability can be subjective/personal, so that one person’s belief and analysis given this belief might differ from that of another person. Bayesian statistics actually is about updating our belief with data. We treat the population parameters as random variables with a defined probability distribution. In this approach we are able to incorporate prior information, i.e. knowledge before having sampled. This approach is also well-suited to updating by repeated sampling. In this paper we show methods using both approaches.

To decide on the number of sampling locations we must first make explicit what quality the result should have. For example, in case of estimating a mean over an administrative unit, we should specify the quality of the estimated mean. This quality can be expressed in various ways. A first option is to express the required quality of the survey result in terms of the maximum standard error of the estimated mean (or areal fraction). A second option is to express the quality in terms of the maximum length of a confidence interval. The length of this interval is proportional to the standard error. We chose this quality criterion to derive the sample size.

In the Bayesian approach the analogue of a confidence interval is a credible interval. A credible interval can be defined in different ways. A highest posterior density (HPD) interval is the credible interval that is a short as possible for a given probability level. For any point inside the interval the density is larger than at any point outside the interval (Lee, 1997). For a unimodal distribution this interval contains the values with the highest probability density, and so includes the mode of the distribution. For a 95% HPD interval with lower bound $b_{l}$ and upper bound $b_{u}$ we believe that there is a 95% chance that the parameter of interest is in the interval ($b_{l},b_{u}$).

### 2.2 Frequentist approach

To estimate a population mean, we assume a normal distribution for the estimated mean. Then the length of a (1-α) confidence interval is

(1) $$l=2\;u_{\left(1-\alpha /2\right)}\frac{\sigma }{\sqrt{n}},$$

where α is the probability that the interval does not contain the population mean, and $u_{\left(1-\alpha /2\right)}$ is the (1-α/2) quantile of the standard normal distribution, and σ is the population standard deviation of the study variable. For example, for a 95% confidence interval $u_{\left(1-\alpha /2\right)}=1.96$. The sample size required for a maximum length of a confidence interval is obtained by rearranging this equation and substituting the prior estimate $\sigma_{0}$ of the population standard deviation σ:

(2) $$n={\left(u_{\left(1-\alpha /2\right)}\frac{\sigma_{0}}{l_{\max}/2}\right)}^{2}\text{.}$$

The parameter $\sigma_{0}$ is referred to as a design parameter, i.e., a parameter that is used to design a sample, in this case to decide on the size of a sample aimed at estimating a population mean.

Various methods are developed for computing a confidence interval of an areal fraction, i.e., the fraction of the area where a condition is (not) met. With simple random sampling this boils down to computing a confidence interval for a binomial probability parameter π. Vollset (1993) compares thirteen methods based on their coverage properties, lengths and errors relative to exact limits. The confidence interval computed by approximating the binomial distribution by a normal distribution is referred to as the Wald confidence interval. With this approximation the length of a (1-α) confidence interval estimate of the areal fraction equals

(3) $$l=2\;u_{\left(1-\alpha /2\right)}\frac{\sqrt{\pi (1-\pi )}}{\sqrt{n-1}}\text{.}$$

Rearranging gives for the sample size

(4) $$n={\left(u_{\left(1-\alpha /2\right)}\frac{\sqrt{\pi_{0}(1-\pi_{0})}}{l_{\max}/2}\right)}^{2}+1,$$

with $\pi_{0}$ a prior estimate of the binomial probability. Note that when designing a sample for estimating an areal fraction, the design parameter is the same as the parameter of interest. For estimating a population mean the design parameter $\sigma_{0}$ differs from the parameter of interest μ.

### 2.3 Bayesian approach

A serious drawback of the frequentist approach for SSD explained in the previous section is that the sample sizes are sensitive to the prior estimates of the design parameters $\sigma_{0}$ and $\pi_{0}$. In general we are rather uncertain about these parameters, and therefore it is attractive to replace single values for these parameters by probability distributions. This leads to a different statistical approach for sample size determination, the Bayesian approach. This approach also offers the possibility of exploiting existing (a priori) information about the population mean or proportion (legacy data), by postulating an informative prior. This informative prior can then be used in SSD, and in a fully Bayesian approach to update the prior once the new data are collected, see hereafter.

The first step in the Bayesian approach of statistical inference is to postulate a prior distribution function for the parameters, the population mean and population standard deviation in case of estimating a mean, and the binomial probability parameter in case of estimating an areal fraction. This function expresses our belief and uncertainty about the parameters before the new sample data are taken into account.

The next step is to formalize a theory about the data. That is, we must assume the type of distribution function of the data, for example a normal or binomial distribution. Once the type of distribution has been specified, we can write an equation for the probability of the data as a function of the distribution parameters. This probability distribution function is referred to as the likelihood function.

The final step is to revise our prior belief about the population parameter of interest, using the data and our theory about the data as expressed in the likelihood function. This results in the posterior distribution function of the parameter. The updated belief is computed with Bayes’ rule:

(5) $$f(\theta |z)=\frac{f(\theta )f(z|\theta )}{f(z)},$$

with

• f(θ|z) the posterior distribution function, i.e., the probability density function of the parameter given the sample data z
• f(θ) our prior belief in the parameter of interest specified by a probability density function
• f(z|θ) the likelihood of the sample data, given values of the distribution parameters θ,
• f(z) the probability distribution function of the sample data.

The posterior distribution for the parameter (Eq. 5) is conditional on data **z**. The problem is that these new data are not yet known. We are designing a sample, and the data are yet to be collected, so at first glance this might seem an unsolvable problem. However, what we could do is to simulate with the prior probability density function a large number of possible vectors with *n* data. In a full simulation approach the following steps are involved:

1. Simulate θ from the prior distribution f(θ)
2. Given the simulated θ, simulate z of length *n* from the model f(z|θ)
3. Given the simulated z calculate the posterior distribution f(θ|z) using Bayes’ rule (Eq. 5)
4. Given the posterior f(θ|z), compute the length of the highest posterior density (HPD) interval with a coverage probability of 1-α, or reversely, the coverage probability of the HPD interval of length $l_{\max}$
5. Repeat steps 1–4 a large number, say *S*, times
6. Compute weighted average of the *S* lengths of the HPD intervals, or the weighted average of the *S* coverage probabilities, using the probability densities of the simulated θ’s as weights

If the average length is larger than $l_{\max}$, or the coverage probability of intervals of length $l_{\max}$ is smaller than 1-α, then we must increase *n*, and if the average length is smaller than $l_{\max}$, or the coverage probability of intervals of length $l_{\max}$ is larger than 1-α, then we must decrease *n* and repeat the whole procedure until our precision requirement is met. Simulation is one option to determine the sample size, (partly) analytical approaches are also available.

More formally, the procedure is as follows. The prior probability density function on the population parameter(s) θ is used to compute for a given sample size *n* the predictive distribution of the data:

(6) $$f(z|n)=\int_{\Theta }f(z|\theta ,n)f(\theta )d\theta$$

with Θ the parameter space for θ containing all possible values of the distribution parameters θ. This predictive distribution is also named the preposterior distribution, stressing that the new data are not yet available.

Even if θ would be fixed, we do not have only one vector z with *n* data values but a probability distribution, from which we can simulate possible data vectors, referred to as the data space Z. In case of a binomial probability and sample size *n*, the data space Z (in the form of the number of observed successes given sample size *n*) can be written as the set {0,1,…,n}, i.e., one vector of length *n* with all failures, *n* vectors of length *n* with one success, n2 vectors with two successes, et cetera. Each data vector is associated with a probability density (for continuous data) or probability mass (for discrete data). As a consequence, we do not have only one posterior distribution function f(θ|z), but as many as we have data vectors in the data space, which is infinitely many in the case of a continuous variable.

Various criteria for SSD can be defined on the basis of all these posteriors, among which are (Joseph et al., 1995, Joseph and Bélisle, 1997)

1. Average length criterion (ALC).
2. Average coverage criterion (ACC).
3. Worst outcome criterion (WOC).

M’Lan et al. (2008) generalized ALC and ACC to criteria based on the length of a credible interval raised to a power *k*, and a median length criterion (MLC) and median coverage criterion (MCC). Here we restrict our analysis to the three criteria listed above.

*Average length criterion.* For a fixed posterior HPD interval coverage of 100(1-α)% the smallest sample size *n* is determined such that

(7) $$\int_{Z}l(z,n)f(z)dz⩽l_{\max},$$

where f(z) is the predictive distribution of the data (Eq. 6), and l(z,n) is the length of the 100(1-α)% HPD interval for data z and sample size *n*, obtained by solving

(8) $$\int_{v}^{v+l(z,n)}f(\theta |z,n)d\theta =1-\alpha ,$$

for l(z,n), for each possible data set z∈Z. f(θ|z,n) is the posterior density of the population parameter of interest given the data z and sample size *n* (Eq. 5). ALC ensures that the average length of 100(1-α)% posterior HPD intervals, weighted by f(z), is at most $l_{\max}$.

*Average coverage criterion.* For a fixed posterior HPD interval of length $l_{\max}$ the smallest sample size *n* is determined such that

(9) $$\int_{Z}\left\{\int_{v}^{v+l_{\max}}f(\theta |z,n)d\theta \right\}f(z)dz⩾1-\alpha ,$$

ACC ensures that the average coverage of HPD intervals of length $l_{\max}$ is at least 1-α. The integral inside the curly brackets is the integral of the posterior density of the population parameter of interest over the HPD interval $\left(v,v+l_{\max}\right)$, given a data vector z of size *n*. The mean of this integrated posterior density of the parameter of interest θ is obtained by multiplying the integrated density with the predictive probability of the data, and integrating over all possible data sets in Z.

*Worst outcome criterion.* Neither ALC nor ACC guarantee that for a particular data set z the criterion is met, as these two criteria are defined as averages over all possible data sets in Z. A more conservative sample size can be computed by requiring that for all data sets Z both criteria are met. Joseph and Bélisle (1997) modified this criterion by restricting the data sets to a subset W of most likely data sets. The criterion thus obtained is referred to as the modified worst outcome criterion, but we will refer to it shortly as the worst outcome criterion. So the criterion is

(10) $$\inf_{z\in W}\left\{\int_{v}^{v+l(z,n)}f(\theta |z,n)d\theta \right\}⩾1-\alpha \text{.}$$

The smallest sample size satisfying this condition is used as the sample size. For instance, if the 95% most likely data sets are chosen as subspace W, WOC guarantees that there is 95% assurance that the length of the 100(1-α)% posterior HPD intervals will be at most $l_{\max}$. The fraction of most likely data sets in subspace W is referred to as the worst level.

### 2.4 Mixed Bayesian-likelihood approach

Besides the fully Bayesian approach, Joseph and Bélisle (1997) describe a mixed Bayesian-likelihood approach for determining the sample size. In this approach the prior is only used to derive the preposterior distribution of the data (Eq. 6), not to derive the posterior of the parameter of interest using Bayes’ rule (Eq. 5). Each sampled data vector is used to derive the posterior using a uniform prior in both the numerator and denominator in Eq. (5). The length of the HPD interval with coverage probability 1-α, or reversely, the coverage probability of the HPD interval of length $l_{\max}$ for a given data vector is then computed from this posterior. This approach is of interest when, after the data have been collected, we prefer to estimate the population mean or areal fraction from these data only, using the frequentist approach described in the previous sections. This may be appropriate if we have doubts about the quality of the legacy data – we are willing to use them to plan the sampling, but not to make statements about the population from which the sample is drawn.

### 2.5 Sample size for estimating a population mean in fully Bayesian and mixed Bayesian-likelihood approach

The three criteria ALC, ACC and WOC are further developed by Adcock (1988) and Joseph and Bélisle (1997) to determine the sample size for estimating a population mean, assuming that the data come from a normal distribution. As we are uncertain about the population standard deviation σ in Eq. (1), a prior distribution is assigned to this parameter. It is convenient and conventional to assign a gamma distribution as a prior distribution to the reciprocal of the population variance, referred to as the precision parameter $\lambda =1/\sigma^{2}$. More precisely, a bivariate prior normal-gamma distribution is assigned to the population mean μ and the precision parameter λ (which is equivalent to a normal-inverse gamma distribution for the mean μ and the variance $\sigma^{2}$):

(11) $$\begin{array}{cc} \lambda \sim & \mathrm{gamma}(a,b)\\ \mu |\lambda \sim & N(\mu_{0},n_{0}\lambda )\text{.} \end{array}$$

with $\mu_{0}$ the mean of the prior distribution for the population mean, and $n_{0}$ the prior sample size. Note that $n_{0}\lambda =\sigma^{2}/n_{0}$, so the variance of the prior for the population mean equals $\sigma^{2}/n_{0}$. In other words $n_{0}$ determines the spread of the prior distribution for the population mean. The larger $n_{0}$, the more squeezed the distribution, the more certain we feel about the population mean. With this prior distribution the predictive distribution of the data is a shifted and scaled *t* distribution with 2a degrees of freedom, with a mean equal to $\mu_{0}$ and a standard deviation (scale) equal to $1/\sqrt{a\;n_{0}/b}$ (Joseph and Bélisle, 1997). For any data vector z, the posterior distribution of the population mean can be computed, which is also a shifted and scaled *t* distribution with known parameters. We refer to Joseph and Bélisle (1997) for these parameters.

The gamma distribution for the precision parameter λ has itself two parameters, *a* and *b*, referred to as hyperparameters. In Section 3.1.1 we explain how these hyperparameters can be set. The mean of a gamma distribution equals a/b, the standard deviation equals $\sqrt{a/b^{2}}$. The sample size using ACC as a criterion can be computed as (Adcock, 1988)

(12) $$n=\frac{4b}{a\;l_{\max}^{2}}t_{2a\text{;}1-\alpha /2}^{2}-n_{0},$$

with $t_{2a\text{;}1-\alpha /2}^{2}$ the squared (1-α/2) quantile of the (usual, i.e., neither shifted nor scaled) *t* distribution with 2a degrees of freedom, and $n_{0}$ the number of prior data.

The prior sample size $n_{0}$ is only relevant if we have prior information about the population mean and an informative normal prior is used for this population mean. If we have no information about the population mean a non-informative prior is used for the population mean and $n_{0}$ equals 0. Note that as a/b is the prior mean of the reciprocal of the population variance $\sigma^{2}$, with $n_{0}=0$ Eq. (12) is similar to Eq. (2). The only difference is that a quantile from the standard normal distribution is replaced by a quantile from a *t* distribution with 2a degrees of freedom.

Joseph and Bélisle (1997) present inequality equations for SSD for ALC and WOC. These complicated equations cannot be solved analytically, but the solution can easily be found by a bisectional search algorithm.

### 2.6 Sample size for estimating a population proportion in fully Bayesian and mixed Bayesian-likelihood approach

The same criteria can be used to estimate the proportion of a population, or in case of an infinite spatial population of points the areal fraction satisfying some condition (Joseph et al., 1995). With simple random sampling this reduces to estimating the probability-of-success parameter π of a binomial distribution. Recall that in this case the space of possible outcomes Z is the number of successes, *z*, which is discrete: Z={0,1,…,n} with *n* the sample size. The conjugate prior distribution for parameter π of the binomial likelihood is the beta distribution:

(13) $$\pi \sim \frac{1}{B(c,d)}\pi^{c-1}{\left(1-\pi \right)}^{d-1},$$

where B(c,d) is the beta function. The beta distribution has two hyperparameters *c* and *d* which correspond to the number of “successes” (1) and “failures” (0) in the problem context. The larger the value of these parameters, the more the prior information, and the more sharply defined the probability distribution. In Section 3.1.2 it is explained how these parameters can be set.

The preposterior marginal distribution of the data is the beta-binomial distribution

(14) $$f(z|n)=\left(\begin{array}{c} n\\ z \end{array}\right)\frac{B(z+c,n-z+d)}{B(c,d)},$$

and for a given number of successes *z* out of *n* trials the posterior distribution of π equals

(15) $$f(\pi |z,n,c,d)=\frac{1}{B(z+c,n-z+d)}\pi^{z+c-1}{\left(1-\pi \right)}^{n-z+d-1}\text{.}$$

For the binomial probability parameter π, criterion ALC (Eq. 7) can be written as

(16) $$\overset{n}{\underset{z=0}{\sum }}l(z,n)f(z|n)⩽l_{\max}\text{.}$$

To determine the smallest *n* satisfying this condition, for each value of *z* (number of successes) and each *n* the length l(z,n) must be computed so that

(17) $$\int_{v}^{v+l(z,n)}f(\pi |z,n,c,d)d\pi =1-\alpha \text{.}$$

with *v* the lower bound of the HPD credible set given the sample size and observed number of successes *z*.

For the binomial probability parameter, criterion ACC (Eq. 9) can be written as

(18) $$\overset{n}{\underset{z=0}{\sum }}\mathrm{Pr}\{\pi \in (v,v+l_{\max})\}f(z|n)⩾1-\alpha ,$$

with

(19) $$\mathrm{Pr}\{\pi \in (v,v+l_{\max})\}\propto \int_{v}^{v+l_{\max}}\pi^{z}{\left(1-\pi \right)}^{n-z}f(\pi )d\pi ,$$

with f(π) the prior density of the binomial probability parameter.

For a binomial probability no closed form formulas exist for SSD. Joseph et al. (1995) describe algorithms for approximating the sample sizes. More recently M’Lan et al. (2008) presented various methods for binomial SSD, among which a method based on a third order approximation, and a Monte Carlo simulation method.

## 3 Case study

As an illustration, we determine the sample sizes for estimating the mean of natural logarithms of Zn within each district and within each mandal of Andhra Pradesh. Previous surveys show that the Zn concentrations within these administrative areas have strong positive skew. Thus assuming a normal distribution of the Zn data is unrealistic. We therefore computed the natural logarithms of the Zn concentrations, and assumed a normal distribution for these transformed data.

We also determined sample sizes for estimating the areal fractions with Zn-deficiency within districts and mandals. This fraction is of practical importance. It can, for example, be used to prioritize districts or mandals for policy interventions. As a critical Zn-concentration, we use 0.9, i.e., if the Zn-concentration at a location is less than 0.9, we consider that this location is deficient of Zn, so that the application of Zn fertilizer is recommended. This threshold is the division between “latent deficiency” and “marginally sufficient” as defined by Shukla and Behera (2019).

In this paper the results for the thirteen districts are presented. The results for all 605 mandals in the state are available at the lead author’s GitHub repository.^2^

The SHC data collected in 2015–2017 (cycle 1) are used to compute the mean and variance of ln(Zn) and the proportion of samples with Zn deficiency per district (Table 1). These legacy sample descriptive statistics are used as prior point estimates of $\sigma_{0}$ (Eq. 2) and $\pi_{0}$ (Eq. 4) for the frequentist approach and to postulate prior distributions for the distribution parameters in the Bayesian and mixed Bayesian-likelihood approach.

**Table 1:** Number of legacy points (n), sample mean of ln(Zn) (μ), sample variance of ln(Zn) ($\sigma^{2}$) and sample proportion with Zn deficiency (π), of cycle 1 SHC data collected in 2015–2017, for districts in Andhra Pradesh, India.

District	n	μ	$\sigma^{2}$	π
Anantapur	49114	−0.73	0.96	0.77
Chittoor	37978	−0.06	0.41	0.49
East Godavari	30353	0.24	0.70	0.33
Guntur	63956	−0.37	0.82	0.61
Kadapa	21739	−0.66	0.60	0.77
Krishna	30481	−0.05	0.80	0.39
Kurnool	79775	−0.39	1.12	0.59
Nellore	48053	−1.22	1.19	0.86
Prakasam	50392	−0.64	1.35	0.67
Srikakulam	40823	0.01	0.44	0.40
Visakhapatnam	8678	−0.41	0.97	0.57
Vizianagaram	28321	−0.35	0.46	0.64
West Godavari	20211	0.37	0.77	0.26

### 3.1 Prior distributions

In the fully Bayesian approach and the mixed Bayesian-likelihood approach uncertainty about the design parameters $\sigma_{0}$ of Eq. (2) and $\pi_{0}$ of Eq. (4) is accounted for by assigning a probability distribution to these parameters.

#### 3.1.1 Gamma distribution for the precision parameter

**Fig. 1:**
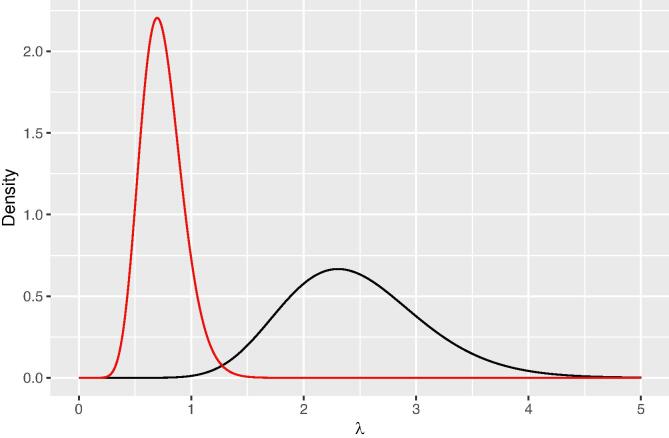
Prior gamma distribution of the precision parameter for Chittoor (black curve) and Prakasam (red curve), for a coefficient of variation of λ of 0.25.

A prior gamma distribution is assigned to the precision parameter $\lambda =1/\sigma^{2}$. The mean of the gamma distribution was set equal to the reciprocal of the legacy sample variance of ln(Zn): $a/b=1/\sigma^{2}$ (Table 1). A second equation with *a* and *b* is needed to derive parameters *a* and *b*. In this second equation the coefficient of variation of the gamma distribution, cv(λ), is set equal to some chosen value expressing how much trust we have in the prior estimate of λ. Solving the two equations with two unknowns gives $a=1/{\left\{\mathrm{cv}(\lambda )\right\}}^{2}$ and $b=a\;\sigma^{2}$. Fig. 1 shows the gamma distributions for the district with the smallest (Prakasam) and largest (Chittoor) value for the precision parameter λ, for a coefficient of variation of 0.25.

**Fig. 2:**
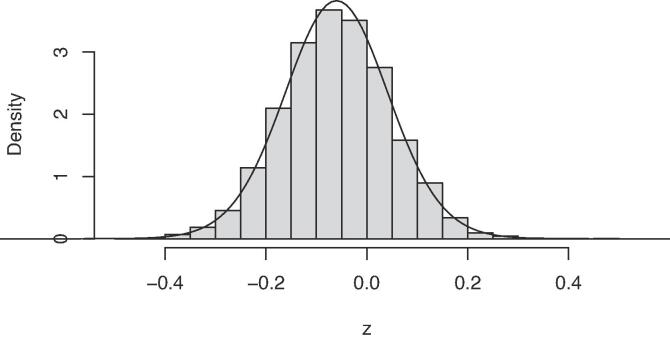
Density plot of 10,000 draws from the prior normal-gamma distribution for the precision parameter and the mean of district Chittoor, and shifted and scaled *t* distribution.

For district Chittoor we drew 10,000 values from the bivariate normal-gamma distribution for the precision parameter and the mean (Eq. 11), see the histogram in Fig. 2. The curve is the density of the shifted and scaled *t* distribution with 2a degrees of freedom, which is the predictive distribution of the ln(Zn) data. The mean of this *t*-distribution is equal to the prior mean $\mu_{0}=-0.06$, the standard deviation of the *t*-distribution equals $1/\sqrt{a\;n_{0}/b}=0.103$. The density curve is not fitted to the histogram, but it is evident that the theoretically-derived density function fits very well the histogram.

#### 3.1.2 Beta distribution for binomial probability parameter

**Fig. 3:**
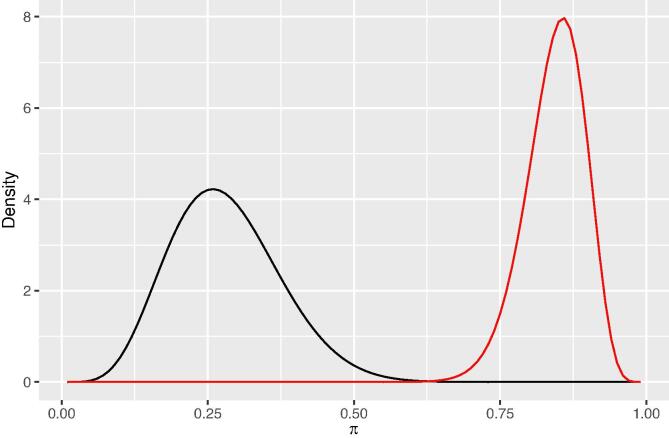
Prior beta distribution of areal fraction with Zn-deficiency, for West Godavari (black curve) and Nellore (red curve).

By setting the mode of the prior beta distribution equal to the legacy sample fraction with Zn deficiency, used as a prior estimate of the areal fraction with Zn deficiency $\pi_{0}$, the parameters of the beta distribution can be computed as (Sambucini, 2017, Eq. 24):

(20) $$\begin{array}{c} c=n_{0}\pi_{0}+1\\ d=n_{0}(1-\pi_{0})+1, \end{array}$$

with $n_{0}$ the prior sample size. The larger $n_{0}$, the larger the values of the parameters, the more sharply defined is the beta distribution, i.e., the more trust we have in the prior estimate of the areal fraction with Zn deficiency. Fig. 3 shows the prior beta distributions for the districts with the smallest (West Godavari) and the largest (Nellore) sample fraction with Zn deficiency: $\pi_{0}=0.259$ and 0.858, respectively. For $n_{0}$ we used the number of legacy data in these districts divided by 1000. This is an arbitrary choice so that the distributions will not be too narrow. The distribution for Nellore is (1) further to the right, i.e., a larger proportion of Zn-deficient observations; (2) sharper than that for West Godavari, because Nellore has many more observations ($n_{0}=48$) than West Godavari ($n_{0}=20$). In both distributions the mode is the most probable value, equal to the legacy sample proportion with Zn deficiency.

**Fig. 4:**
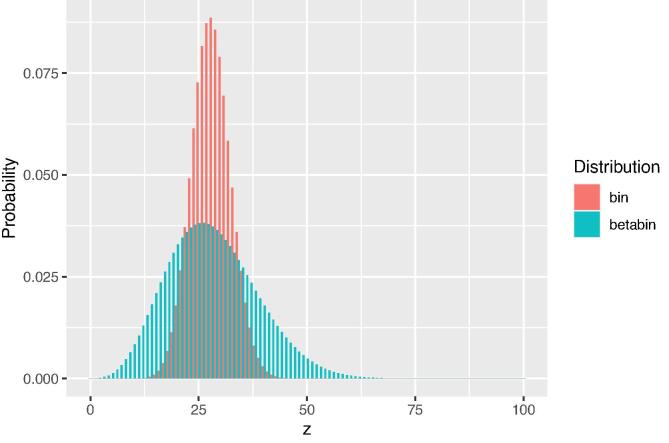
Beta-binomial predictive distribution for West Godavari for a sample size of 100. For comparison the binomial distribution is plotted with a probability parameter equal to c/(c+d) of the prior beta distribution.

Fig. 4 shows the beta-binomial predictive distribution of the data, for West Godavari, for a sample size of 100. For comparison we also plotted the binomial distribution for the same sample size and a binomial probability parameter equal to c/(c+d) of the prior beta distribution (*c* and *d* computed with $n_{0}=20$, Eq. (20)). With increasing $n_{0}$ the beta-binomial distribution approaches the binomial distribution.

### 3.2 Required sample sizes

#### 3.2.1 Mean of ln(Zn)

Table 2 shows the sample sizes for credible (confidence) intervals of (average) length 0.2 and (average) coverage of 95%. For WOC with the fully Bayesian and the mixed Bayesian-likelihood approach 80% of the most likely data sets are used (worst level is 80%). For the Bayesian approach we used an uninformative flat prior for the mean of ln(Zn), or more precisely stated, a normal distribution with an infinitely large variance, so $n_{0}=0$ (Eq. 11). The sample size as determined with the frequentist approach ranges from 157 for district Chittoor, which has the smallest value for $\sigma_{0}^{2}$ (Table 1) (and so the largest value for the prior mean of λ), to 517 points for district Prakasam which has the largest value for $\sigma_{0}^{2}$. The fully Bayesian sample sizes are larger than the frequentist sample sizes. For ALC, the increase of the sample size is about 5% of the frequentist sample size, for ACC this increase is about 8%, and for WOC about 26%. The mixed Bayesian-likelihood sample sizes are slightly larger than the fully Bayesian sample sizes. For ALC, the difference is only one point (except for Anantapur with a difference of two points, which is most likely an approximation error), showing that the information in an uninformative, uniform prior for the mean is one point. Recall that when using an informative prior for the mean, a prior normal distribution with precision $n_{0}\lambda$, all three fully Bayesian sample sizes are reduced by $n_{0}$ points, so that they can become smaller than the frequentist sample sizes. The sample sizes computed with $n_{0}=0$ are conservative estimates of the sample size with the Bayesian approach.

**Table 2:** Frequentist, fully Bayesian and mixed Bayesian-likelihood (mbl) sample sizes required for a confidence (credible) interval of (average) length 0.2 and an (average) coverage of 95% for the population mean of ln(Zn). For WOC the 80% most likely data sets are used. The fully Bayesian and mixed Bayesian-likelihood sample sizes are computed with a prior gamma distribution for λ with a coefficient of variation of 0.25. The fully Bayesian sample sizes are for a prior sample size of zero ($n_{0}=0$).

District	λ	Freq	ALC	ALC(mbl)	ACC	ACC(mbl)	WOC	WOC(mbl)
Anantapur	1.05	368	386	388	397	399	466	472
Chittoor	2.46	157	165	166	169	171	197	204
East Godavari	1.44	268	282	283	289	292	339	346
Guntur	1.23	314	330	331	339	342	398	403
Kadapa	1.67	231	243	244	249	251	292	298
Krishna	1.25	307	323	324	331	335	388	395
Kurnool	0.89	432	454	455	467	470	548	555
Nellore	0.84	458	482	483	495	498	581	589
Prakasam	0.74	517	544	545	559	561	656	662
Srikakulam	2.29	168	177	179	182	185	212	218
Visakhapatnam	1.03	372	391	392	402	404	471	478
Vizianagaram	2.17	177	187	188	191	194	223	231
West Godavari	1.31	295	310	311	318	321	373	380

Recall that the credible (confidence) intervals are on the log-scale. After back-transformation the length of the interval is not constant, but depends on the mean of ln(Zn). The smaller this mean, the shorter the length. The length after back-transformation, $l^{*}$, is proportional to exp(μ): $l^{*}=l\;\exp(\mu )$ (*l* is 0.2 in our case).

**Fig. 5:**
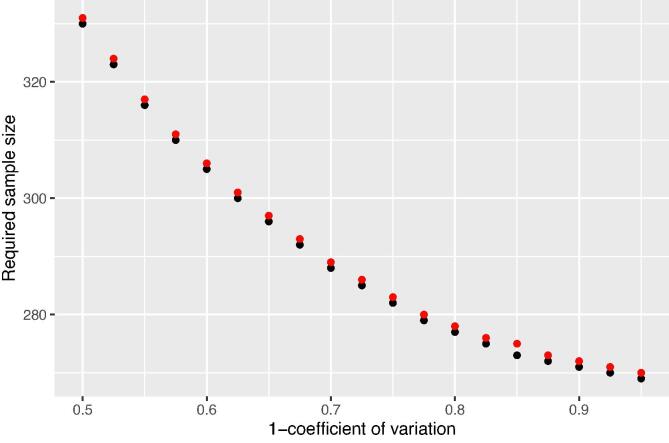
Effect of coefficient of variation of the prior gamma distribution of the precision parameter on the fully Bayesian (black dots) and mixed Bayesian-likelihood (red dots) sample sizes, using ALC as a criterion, for a credible interval of average length 0.2 and a coverage of 95% for the mean of ln(Zn) of East Godavari.

Fig. 5 shows the effect of the coefficient of variation of the gamma distribution for the precision parameter on the sample sizes for district East Godavari, using ALC as a criterion. Note that we plotted the complement of the coefficient of variation on the x-axis, so that the prior becomes more informative along this axis. The smaller the coefficient of variation (the larger the complement), the less uncertain we are about the precision parameter, the smaller the sample size. With decreasing uncertainty about the precision parameter (population variance parameter), the fully Bayesian and mixed Bayesian-likelihood sample size as determined with ALC and ACC asymptotically approach the frequentist sample size (which is 268 for East Godavari, Table 2). With $n_{0}>0$ the Bayesian sample size then is $n_{0}$ points smaller than the frequentist sample size.

**Fig. 6:**
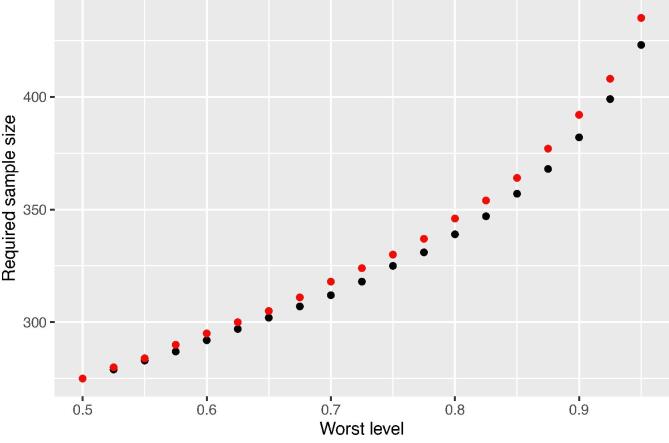
Effect of worst level on the fully Bayesian (black dots) and mixed Bayesian-likelihood sample sizes (red dots), for a 95% credible interval of length 0.2 for the mean of ln(Zn) of East Godavari. Sample sizes are determined with a prior gamma distribution for λ with a coefficient of variation of 0.25. The prior sample size in the fully Bayesian approach is zero ($n_{0}=0$).

Fig. 6 shows the effect of the worst level on the sample sizes for district East Godavari using a prior gamma distribution for λ with a coefficient of variation of 0.25. The more certain we want to be that for an individual sample (i.e., a random sample with the size set by this analysis) the length of the 95% credible interval of ln(Zn) does not exceed 0.2, the larger the sample size. For a worst level of 0.5 the sample sizes are 275 and 276 for the fully Bayesian and mixed Bayesian-likelihood approach, respectively. These sample sizes are slightly smaller than the sample sizes determined with ALC (Table 2 282 and 283 points). The same relative difference was observed for the other districts. This shows that in this case the required samples sizes determined with ALC assures that in a bit more than 50% of the samples the (1-α)% credible interval does not exceed the length $l_{\max}$.

#### 3.2.2 Areal fraction with Zn deficiency

Table 3 shows the sample sizes for credible intervals (confidence intervals) of (average) length 0.1 and (average) coverage of 95%. The parameters of the beta distribution for the binomial probability parameter (Eq. 20) are computed with a prior sample size $n_{0}$ equal to the number of legacy points divided by 1000 (Table 1). As before, for WOC a worst level of 80% is used, i.e., 80% of the most likely data sets are used to determine the sample sizes. The sample sizes with the fully Bayesian approach are smaller than the frequentist sample sizes. The mixed Bayesian-likelihood sample sizes using ALC and ACC as a criterion are about equal to the frequentist sample sizes (for some districts some what smaller, for other districts somewhat larger).

**Table 3:** Frequentist (Wald), fully Bayesian and mixed Bayesian-likelihood (mbl) sample sizes required for a credible interval of (average) length 0.1 and an (average) coverage of 95% for the areal fraction with Zn deficiency. The prior sample size $n_{0}$ is equal to the number of legacy points divided by 1000. For WOC the 80% most likely data sets are used.

District	π	Wald	ALC	ALC(mbl)	ACC	ACC(mbl)	WOC	WOC(mbl)
Anantapur	0.77	275	223	271	226	276	263	318
Chittoor	0.49	386	335	371	335	371	343	381
East Godavari	0.32	339	299	327	301	330	336	368
Guntur	0.61	366	294	356	295	357	311	376
Kadapa	0.77	272	249	269	256	278	310	335
Krishna	0.39	368	324	353	325	354	348	378
Kurnool	0.59	373	286	364	286	364	298	379
Nellore	0.86	189	144	192	149	201	187	247
Prakasam	0.67	340	282	331	284	333	310	364
Srikakulam	0.40	371	319	358	319	359	337	379
Visakhapatnam	0.57	377	333	340	337	345	370	378
Vizianagaram	0.64	355	315	341	317	344	347	375
West Godavari	0.26	296	271	289	277	297	328	352

The sample size according to the frequentist approach is the largest for the district Chittoor. For this district the sample proportion with Zn deficiency, used as a prior estimate of the areal fraction with Zn deficiency, $\pi_{0}$, equals 0.495, i.e., close to 0.5. The closer the sample proportion to 0 or 1, the smaller the sample size. This is easily understood, as the population variance, equal to π(1-π), is maximal for an areal fraction of 0.5. Also with the fully Bayesian approach and the mixed Bayesian-likelihood approach with all three criteria this district requires the largest sample size.

**Fig. 7:**
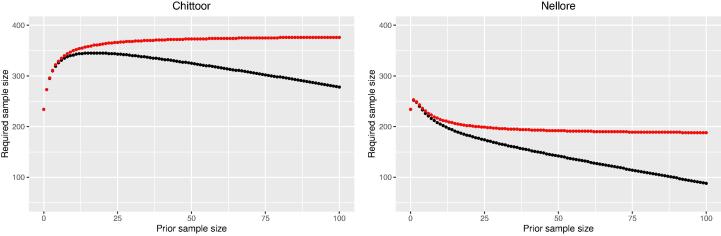
Effect of prior sample size on the fully Bayesian (black dots) and mixed Bayesian-likelihood sample sizes (red dots), using ALC as a criterion, for a credible interval of average length 0.1 and a coverage of 95% for the areal fraction with Zn deficiency in Chittoor and Nellore.

Fig. 7 shows the effect of the prior sample size $n_{0}$ on the sample size for districts Chittoor and Nellore, using ALC as a criterion. For Nellore with a $\pi_{0}$ of 0.86 both the fully Bayesian and the mixed Bayesian-likelihood sample sizes decrease with $n_{0}$, except that for $n_{0}=0$ the sample size is smaller than for $n_{0}=1$. With increasing $n_{0}$ the three fully Bayesian sample sizes go to zero. The mixed Bayesian-likelihood sample sizes as obtained with ALC and ACC asymptotically approach the frequentist sample sizes.

For Chittoor a different effect can be seen. The mixed Bayesian-likelihood sample size asymptotically increases with $n_{0}$, whereas the fully Bayesian sample size first increases and reaches a maximum of 345 points at $n_{0}=14$, remains stable until $n_{0}=21$, and than decreases again. For small $n_{0}$ the prior beta distribution of $\pi_{0}$ is very flat (Eq. 20). With this prior distribution data vectors with small and high sample fractions with Zn deficiency are simulated, as well as data vectors with sample fractions close to 0.5. With increasing $n_{0}$ the probability mass around the prior estimate $\pi_{0}$ increases. For Chittoor this prior estimate equals 0.49 (Table 3). With increasing $n_{0}$ more data vectors with sample fractions close to 0.49 are simulated. In the mixed Bayesian-likelihood approach the confidence interval is fully based on the likelihood of the data. With a sample fraction equal to 0.5 the length of a confidence interval (for a given α) is maximal. This explains that for large $n_{0}$, leading to a prior distribution with most probability mass around 0.49, the required sample size computed wit ALC is largest. The maximum required sample size with the mixed Bayesian-likelihood approach equals 376 points, which is somewhat smaller than the frequentist sample size of 386 points based on the Wald confidence interval (Table 3). The difference is likely caused by the different approximation of the length of a confidence interval in the software used for computing the mixed Bayesian-likelihood sample sizes.

As opposed to the mixed Bayesian-likelihood approach in the fully Bayesian approach the prior distribution of the areal fraction is also used to update this prior to a posterior. The larger $n_{0}$, the stronger our belief in this prior. This strong belief may be based on a large number of existing observations (large legacy sample). So with increasing $n_{0}$ more data vectors with sample fractions close to 0.49 are simulated, which would lead to an increase of the fully Bayesian sample size, but we give more weight on the prior, leading to a decrease of the sample size. Apparently until $n_{0}=14$ the first effect dominates, then both effects are in balance, and beyond $n_{0}=21$ the second effect dominates.

For all districts the effect of $n_{0}$ on the mixed Bayesian-likelihood sample sizes is strong for small prior sample sizes, but then levels off rapidly. The prior sample size at which the effect levels off varies from three points (Anantapur) to about 50 points (Krishna, Krnool, Prakasam), see supplementary material.

For $n_{0}=0$ the fully Bayesian and mixed Bayesian-likelihood sample sizes are equal for all districts, and the mixed Bayesian-likelihood sample sizes are equal to the fully Bayesian sample sizes. The sample sizes are 234, 274 and 366 for ALC, ACC and WOC respectively. The frequentist sample sizes are independent of $n_{0}$, and remain unchanged (Table 3). For $n_{0}=0$ both parameters *c* and *d* of the beta distribution are 1, and the prior distribution of the binomial probability parameter is a uniform distribution, see Eq. (13). The sample size is thus determined for any value of π, not just for a single value of π equal to the legacy sample proportion as in the frequentist approach. With a uniform prior distribution of π, the beta-binomial preposterior distribution of the data (Eq. 14) is also a uniform distribution: all values of *z* (0,1,…,n) have equal probability.

**Fig. 8:**
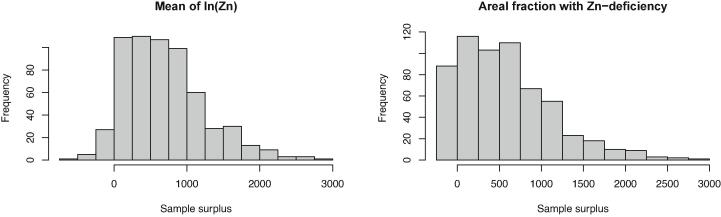
Surplus of sampling points at the mandal level, computed as the mixed Bayesian-likelihood sample sizes using ALC as a criterion minus the current sample size in SHC survey, for the mean of ln(Zn) and the areal fraction with Zn-deficiency.

Though we acknowledge that the SHC scheme is oriented towards field management, we have shown that for district-level assessments all sample sizes are substantially smaller than the current sample sizes applied in the SHC scheme (Table 1). Even at the mandal level, for most mandals the required sample sizes are smaller than the current sample sizes. Fig. 8 shows the surplus of sampling points at the mandals level, for the mixed Bayesian-likelihood approach, using ALC as a criterion and the same precision requirements as before. Only for a few mandals is the required sample size larger than the current sample size.

## 4 Discussion

We are always uncertain about the design parameters (i.e., a parameter that is used to design a sample) σ and π, and therefore it is reasonable to account for this uncertainty in determining the sample size. In Bayesian SSD this is accomplished by postulating a prior distribution for the parameter. As shown in the case study, the sample size with the fully Bayesian and mixed Bayesian-likelihood approach are sensitive to the prior distributions of the parameters. Specifically, for a population mean the two parameters of the gamma distribution for the precision parameter, and for an areal fraction the two parameters of the beta distribution for the binomial probability parameter have a strong effect on the sample size.

Because of this sensitivity ample attention should be paid to the choice of these hyperparameters of the prior distribution. In our case study we derived the parameters of the beta distribution from the legacy sample proportion with Zn-deficiency and an arbitrary choice of the prior sample size $n_{0}$, computed by multiplying the legacy sample size by 0.001. The hyperparameters of the gamma distribution were derived from the sample variance of ln(Zn) and an arbitrary choice on the coefficient of variation of the gamma distribution.

Another option, especially applicable in the absence of legacy data, would have been expert elicitation. Although expert knowledge is subjective, it is based on experience and knowledge of the study area, and thus is likely better than ignorance or arbitrary choices. In case we have legacy data from the study area, experts may also help to choose a prior distribution. How much trust do experts have in a prior estimate of the mean or areal fraction as computed from the legacy data? If the confidence of an expert in this prior estimate is expressed in terms of an interval, this interval can be used to derive the hyperparameters. For instance, if according to an expert the areal fraction with Zn deficiency for East Godavari is most likely between 0.25 and 0.5, the hyperparameters of the beta distribution are 21 and 35, interpreting the limits as the 2.5% and 97.5% quantiles of this distribution. This corresponds with a prior sample size of 56 points (Eq. 20). This leads to a Bayesian sample size of 241 points and a mixed Bayesian-likelihood sample size of 349 points for an average length of a 95% credible interval of 0.1.

If an expert believes that most likely the population variance of ln(Zn) in district East Godavari is between 0.60 and 0.90, the hyperparameters of the gamma distribution for the precision are 94 and 68, leading to an upper bound of the Bayesian sample size ($n_{0}=0$) of 281 points, and a mixed Bayesian-likelihood sample size of 283 points for an average length of a 95% credible interval of 0.2.

An alternative approach is not to use a single prior, but a class of plausible priors, to explore the variation of the criterion used for SSD (e.g. ALC) due to uncertainty about the prior. This leads to robust Bayesian SSD, see for instance De Santis (2006) and Brutti et al. (2008). In this context it is also worth noting that there is no need that the same prior is used for computing the predictive distribution of the data and for the analysis of the posterior distribution given a vector with data. In the two-priors approach for Bayesian SSD the design prior may differ from the analysis prior, which is more flexible (Brutti et al., 2014). In essence, the mixed Bayesian-likelihood approach can also be seen as a two-priors Bayesian approach, in which the analysis prior is a non-informative uniform prior, whereas the design prior can be an informative prior.

The fully Bayesian and mixed Bayesian-likelihood approach for SSD is of specific interest for an adaptive sampling approach in which soil data are collected in phases. The data of each phase are used to derive a prior distribution for the design parameter(s) which is then used to determine the required sample size of subsequent phase. This has features in common with the adaptive Bayesian approach for SSD of a reconnaissance survey aimed at estimating a variogram proposed by Marchant and Lark (2006). This variogram is needed to derive the required spacing of a sampling grid for mapping given a threshold for the maximum kriging variance.

The starting point in determining the sample size is the choice of the “confidence” level α and the maximum length of the credible interval $l_{\max}$. Decreasing α and/or $l_{\max}$ will lead to a larger sample size, and vice versa. So the question is what is a reasonable choice for these two parameters. Increasing the sample size results in a more precise estimate of the population mean of Zn and areal fraction with Zn-deficiency, but also in higher costs. Does this pay? For instance, should we collect more data to decide on the application of Zn-fertilizer in a district or mandal? Through collecting additional data, we are less uncertain, and as a result the probability of a wrong decision becomes smaller. But what is the monetary value of this? In this example, the expected value of the information (VOI) is the reduced probability of a wrong decision, multiplied by the average consequence of being wrong. This expected VOI is then compared with the costs of collecting the additional data. A recent example of this VOI approach applied on soil carbon monitoring is de Gruijter et al. (2016). In a Bayesian framework this is know as the maximization of the expected utility (MEU) approach (Lindley, 1997), which is contrasted with a performance based approach applied in this research.

It is evident that Zn is not the only soil fertility parameter of interest. The main aim of this paper is to show how the sample size can be determined under a Bayesian approach. To decide on the ultimate sample size this approach should also be applied for other crucial soil fertility parameters. The maximum of the sample sizes over these soil fertility parameters can then be used as the ultimate sample size. An alternative approach is to use as the study variable the output of a soil-crop simulation model that integrates all relevant soil fertility parameters, and to determine the sample size for estimating the mean of the model output.

As mentioned in the Introduction the SHC survey data primarily aims at addressing production challenges at the level of individual fields though soil maps at district level are developed as well. The data can also be used to develop more granular (gridded) maps of the soil fertility parameters with digital soil mapping (DSM), so that we have an estimate of the soil fertility parameters at any location in the study area, and so for any individual field. In other words, SHC aims at answering questions at multiple spatial scales: districts, mandals and individual fields. To serve these different aims we search for a sampling design type that is efficient both for estimating means and areal fractions of districts (mandals), and for DSM. The efficiency of a sampling design type for DSM largely depends on the mapping method (Brus, 2019). Mapping methods that exploit the availability of maps of covariates related to the soil properties of interest, such as terrain attributes, climate variables and variables derived from remote sensing imagery, are most promising. For these mapping methods spreading of the sampling locations in feature space may increase the efficiency. To ensure that the same data can also be used for design-based estimation of means and areal fractions of districts and mandals, we propose to select the sampling locations by probability sampling, using a design type that results in samples that are well-spread in the space spanned by important features. An interesting sampling design type for this is the local pivotal method (Grafström et al., 2012, Grafström and Tillé, 2013). In further research we will analyze how many data are needed for taking decisions on fertilization at the level of individual fields.

The Soil Health Card survey is designed as a monitoring project: every two years the fertility of the soil is surveyed. Besides interest in the current status of soil fertility parameters, users are also interested in changes of the soil fertility parameters over time. An interesting question in the context of this paper is, for instance, how precise the change in the mean of Zn and the areal fraction with Zn deficiency of a district can be estimated with the sample sizes reported in this paper. In a frequentist approach the variance of the estimated change depends on the space–time design (de Gruijter et al., 2006). It is well-known that revisiting the same locations of the first sampling round in the second sampling round results in the most precise estimate of the change of the estimated mean and areal fraction. But for estimating the current status, replacing a proportion of the sampling locations by new locations can be more efficient. The optimal proportion depends on the correlation of the two measurements at the same location (paired data) (de Gruijter et al., 2006, section 15.2.3). Optimal sampling design for monitoring soil fertility is the central topic of further research.

## 5 Conclusions

In SSD uncertainty about the parameter of interest such as the population mean or areal fraction can nicely be accounted for in a Bayesian approach.

With the priors chosen in this paper the fully Bayesian and mixed Bayesian-likelihood sample sizes are comparable with the frequentist sample sizes based on the average length (ALC) or average coverage (ACC) of the credible interval. When the worst outcome criterion is used, these sample sizes are larger than the frequentist sample sizes, depending on the worst level (proportion of most likely data sets). However, the fully Bayesian sample sizes for the population mean are conservative, assuming a prior sample size of zero points. With more realistic prior sample sizes the fully Bayesian sample size can become substantially smaller than the frequentist sample size.

The fully Bayesian and mixed Bayesian-likelihood sample sizes are sensitive to the hyperparameters of the prior distributions. The coefficient of variation of the gamma distribution for the precision parameter had a strong effect on the sample size (Fig. 5). For the areal fraction with Zn deficiency the effect of the prior sample size (used to compute the hyperparameters of the beta distribution for the binomial probability parameter) on the mixed Bayesian-likelihood sample sizes is strong for small prior sample sizes, but then levels off rapidly. The prior sample size at which the effect levels off varies from three points to about fifty points. At the district level all sample sizes are much smaller than the current sample sizes used in the SHC surveys. Even at the mandals level for nearly all mandals the current sample sizes are in excess of the Bayesian and mixed Bayesian-likelihood sample sizes.

A sample survey is performed in order to provide information for decision makers. Whether the SSD methods are sophisticated or simple, the challenge is to explain to the decision makers, who finance the survey, not only the recommended sample size, but some idea on how these were computed. This communication begins already when determining the precision with which they require information, and it is hoped that the trust between statisticians and their clients can be established throughout the survey process.

## Software

Package SampleSizeMeans (Joseph and Bélisle, 2012) is used to determine Bayesian sample sizes for normal means, for the fully Bayesian and the mixed Bayesian-likelihood approach.

Sample sizes using the fully Bayesian and the mixed Bayesian-likelihood approaches for binomial probabilities (areal fractions) are computed with R package SampleSizeBinomial, available at http://www.medicine.mcgill.ca/epidemiology/Joseph/software/Bayesian-Sample-Size.html.

Sample sizes for estimating an areal fraction using the frequentist approach are computed with R package binomSamSize (Höhle, 2017). This package has quite a few functions for computing the sample size. The function ciss.wald uses the normal approximation.

The parameters of the beta distribution, given the limits of an interval for the binomial probability, were computed with R function beta.parms.fromquantiles.R, available at http://www.medicine.mcgill.ca/epidemiology/Joseph/pbelisle/BetaParmsFromQuantiles.html.

The parameters of the gamma distribution, given the limits of an interval for the precision parameter, are computed with R function gamma.parms.fromquantiles.R, available at http://www.medicine.mcgill.ca/epidemiology/Joseph/pbelisle/BetaParmsFromQuantiles.html.

## Declaration of Competing Interest

The authors declare that they have no known competing financial interests or personal relationships that could have appeared to influence the work reported in this paper.

## Appendix A Supplementary material

The R script and the data can be downloaded from https://github.com/DickBrus/RequiredSampleSizes4AndhraPradeshDistricts

## Footnotes

1 https://soilhealth7.gov.in/

2 https://www.github.com:DickBrus/RequiredSampleSizes4AndhraPradeshDistricts.git.
